# Simulated lunar microgravity transiently arrests growth and induces osteocyte-chondrocyte lineage differentiation in human Wharton’s jelly stem cells

**DOI:** 10.1038/s41526-024-00397-1

**Published:** 2024-05-04

**Authors:** Arjunan Subramanian, Chelsea Han Lin IP, Wei Qin, Xiawen Liu, Sean W.D. Carter, Gokce Oguz, Adaikalavan Ramasamy, Sebastian E. Illanes, Arijit Biswas, Gabriel G. Perron, Erin L. Fee, Sarah W. L. Li, Michelle K.Y. Seah, Mahesh A. Choolani, Matthew W. Kemp

**Affiliations:** 1 Department of Obstetrics and Gynaecology, NUS Yong Loo Lin School of Medicine, National University of Singapore, 1E Kent Ridge Road, NUHS Tower Block, Level 12, Singapore, 119228 Singapore; 2 Department of Obstetrics and Gynecology, Nanxishan Hospital of Guangxi Zhuang Autonomous Region, No. 46 Chongxin Road, 541002 Guilin City, Guangxi Zhuang Autonomous Region P. R. China; 3 Guangzhou Municipal and Guangdong Provincial Key Laboratory of Molecular Target & Clinical Pharmacology, The NMPA and State Key Laboratory of Respiratory Disease, School of Pharmaceutical Sciences and the Fifth Affiliated Hospital Guangzhou Medical University, 511436 Guangzhou, P.R. China; 4 Genome Institute of Singapore (GIS). Agency for Science, Technology and Research (A*STAR), 60 Biopolis Street, Genome #02-01, Singapore, 138632 Republic of Singapore; 5 Department of Obstetrics and Gynecology, Faculty of Medicine, Universidad de los Andes, Santiago, 7620001 Chile; 6 IMPACT, Center of Interventional Medicine for Precision and Advanced Cellular Therapy, Santiago, Chile; 7 Department of Obstetrics and Gynaecology, National University Hospital, 1E Kent Ridge Road, NUHS Tower Block, Level 12, Singapore, 119228 Singapore; 8 Center for Genomics and Systems Biology, New York University, New York, NY 10003 USA; 9 Division of Obstetrics and Gynaecology, University of Western Australia, Perth, WA Australia; 10 Women and Infants Research Foundation, King Edward Memorial Hospital, Subiaco, WA Australia; 11 Centre for Perinatal and Neonatal Medicine, Tohoku University Hospital, Sendai, 980-8574 Japan

**Keywords:** Stem cells, Cancer stem cells

## Abstract

Human Wharton’s jelly stem cells (hWJSCs) are multipotent stem cells that are extensively employed in biotechnology applications. However, the impact of simulated lunar microgravity (sμG) on the growth, differentiation, and viability of this cell population is incompletely characterized. We aimed to determine whether acute (72 h) exposure to sμG elicited changes in growth and lineage differentiation in hWJSCs and if putative changes were maintained once exposure to terrestrial gravity (1.0 G) was restored. hWJSCs were cultured under standard 1.0 G conditions prior to being passaged and cultured under sμG (0.16 G) using a random positioning machine. Relative to control, hWJSCs cultured under sμG exhibited marked reductions in growth but not viability. Cell population expression of characteristic stemness markers (CD 73, 90, 105) was significantly reduced under sμG conditions. hWJSCs had 308 significantly upregulated and 328 significantly downregulated genes when compared to 1.0 G culture conditions. Key markers of cell replication, including *MKI67*, were inhibited. Significant upregulation of osteocyte–chondrocyte lineage markers, including *SERPINI1, MSX2, TFPI2, BMP6, COMP, TMEM119, LUM, HGF, CHI3L1* and *SPP1*, and downregulation of cell fate regulators, including *DNMT1* and *EZH2*, were detected in sμG-exposed hWJSCs. When returned to 1.0 G for 3 days, sμG-exposed hWJSCs had accelerated growth, and expression of stemness markers increased, approaching normal (i.e. 95%) levels. Our data support earlier findings that acute sμG significantly reduces the cell division potential of hWJSCs and suggest that acute sμG-exposure induces reversible changes in cell growth accompanied by osteocyte–chondrocyte changes in lineage differentiation.

## Introduction

Wharton’s jelly is a mucoid connective tissue principally comprised of stem cells, collagens, and proteoglycan^1^. Covered by the amniotic epithelium and enveloping the vessels (arteries, veins, urachus) of the umbilical cord, Wharton’s jelly provides structural support and reduces the risk of vessel occlusion during fetal growth. Human Wharton’s jelly stem cells (hWJSCs) are abundant and primarily located in the perivascular region of the umbilical cord, offering a convenient source for research and biotechnological applications^2^.

These cells are classified as mesenchymal stem cells (MSCs) because they have the typical adherent characteristics of MSC, they have the accepted CD markers, and the ability to differentiate into osteocytes, adipocytes, and chondrocytes in vitro^3^. hWJSCs from a variety of species have been shown to express several embryonic stem cell markers (*POU5F1*, *NANOG*, *SOX2*, *CKIT*, *DNMT3B*), and they have immunomodulatory properties that are of particular interest for wound healing and tissue regeneration studies^4–6^. These cells possess a more immature phenotype, high plasticity, morphology retention during prolonged in vitro passages, and sustained immunoprivilege, especially when compared to other types of MSCs^7^.

A number of investigators have reported methods for the differentiation of hWJSCs into endodermal (i.e., hepatocytes, pancreatic islet-like cells), mesodermal (skeletal and cardiac muscle, osteoblasts and chondrocytes, adipocytes, endothelial cells) and ectodermal (neurons) lineages^4–6^. Mitchell et al. reported that hWJSCs can be induced towards neuronal lineage following treatment using basic fibroblast growth factor, butylated hydroxyanisole and dimethylsulfoxide in a low-serum media. Just several hours post-induction resulted in the expression of a neural stem cell marker neuron-specific enolase. Whereas overnight treatment resulted in neural stem cell-like morphological changes, and further treatment up to 72 h caused hWJSCs to resemble primary neuron cultures, expressing additional neuronal markers including class III β-tubulin, neurofilament M, and tyrosine hydroxylase^8^. Conconi and colleagues used histological approaches to show the induction of myogenic (*MYF5*, *MYOD*), osteogenic (mineralized matrix deposition) and adipogenic capacities (Oil Red O positive) at seven and fourteen days of culture in differentiation media^9^. Similarly, Zhang et al. reported the expression of hepatocyte lineage markers in hWJSCs after 7 days of culture with hepatocyte growth factor and fibroblast growth factor^10^. Additional studies have explored the effects of altered oxygen tension, shear stress, and low-serum conditions on differentiation capacity and growth^11^.

In contrast, there are comparably fewer studies that describe the impact of sμG on hWJSCs and whether this approach may be used to drive lineage differentiation. Pala and colleagues assessed an a priori selected set of stemness, senescence, and oxidative stress markers over a 48-h sμG experiment, reporting reductions in *NANOG* and *SOX2* expression after the 6- h time point^7^, with other studies showing down-regulation of cell cycle proteins in association with reduced growth after 72 h of sμG culture^12^. Experiments using limbal fibroblasts from corneal transplant tissue reported reductions in cell growth after 48 h of rotary cell culture, with increased expression of mesenchymal stem cell markers^13^.

The data presented in each of the above studies suggests that, at a transcriptional level, lineage-altering changes should be rapidly detectable in hWJSCs following the introduction of sμG exposure. To assess the potential of sμG exposure to induce lineage differentiation in hWJSCs, we correlated growth, cell marker expression, and viability with bulk RNA transcriptional and immunohistochemical changes in seven primary hWJSCs lines. Anticipating changes in lineage differentiation and growth characteristics, we examined the maintenance of these alterations following the return of these hWJSCs lines to normal gravity. Our data show that acute, 3-day exposure to sμG elicits reductions in growth potential concomitant with mesodermal lineage changes consistent with osteocyte–chondrocyte differentiation. Interestingly, when returned to 1.0 G, growth and stemness marker changes were lost, but alterations in transcriptional lineage changes were broadly maintained. Moreover, when cultured for 14 days in either osteocyte or chondrocyte lineage differentiation media, hWJSCs exposed to sμG had significant increases in staining for osteocyte and chondrocyte differentiation relative to sμG-naïve control cells.

Data from earlier studies strongly indicate that lineage-altering changes should be rapidly detectable at the transcriptional level in hWJSCs following exposure to sμG. To evaluate the potential of sμG exposure to induce lineage differentiation in hWJSCs, we conducted a comprehensive analysis that correlated growth, cell marker expression, and viability with bulk RNA transcriptional changes across seven primary hWJSC lines. In anticipation of observing changes in lineage differentiation and growth characteristics, we also investigated the persistence of these alterations after these hWJSC lines returned to normal gravity conditions. We aimed to evaluate the influence of sμG on growth potential along with mesenchymal lineage changes, as well as the restoration of basal cell characteristics upon re-entry into 1.0 G conditions.

## Results

### Cell attachment and morphology

**Fig. 1:**
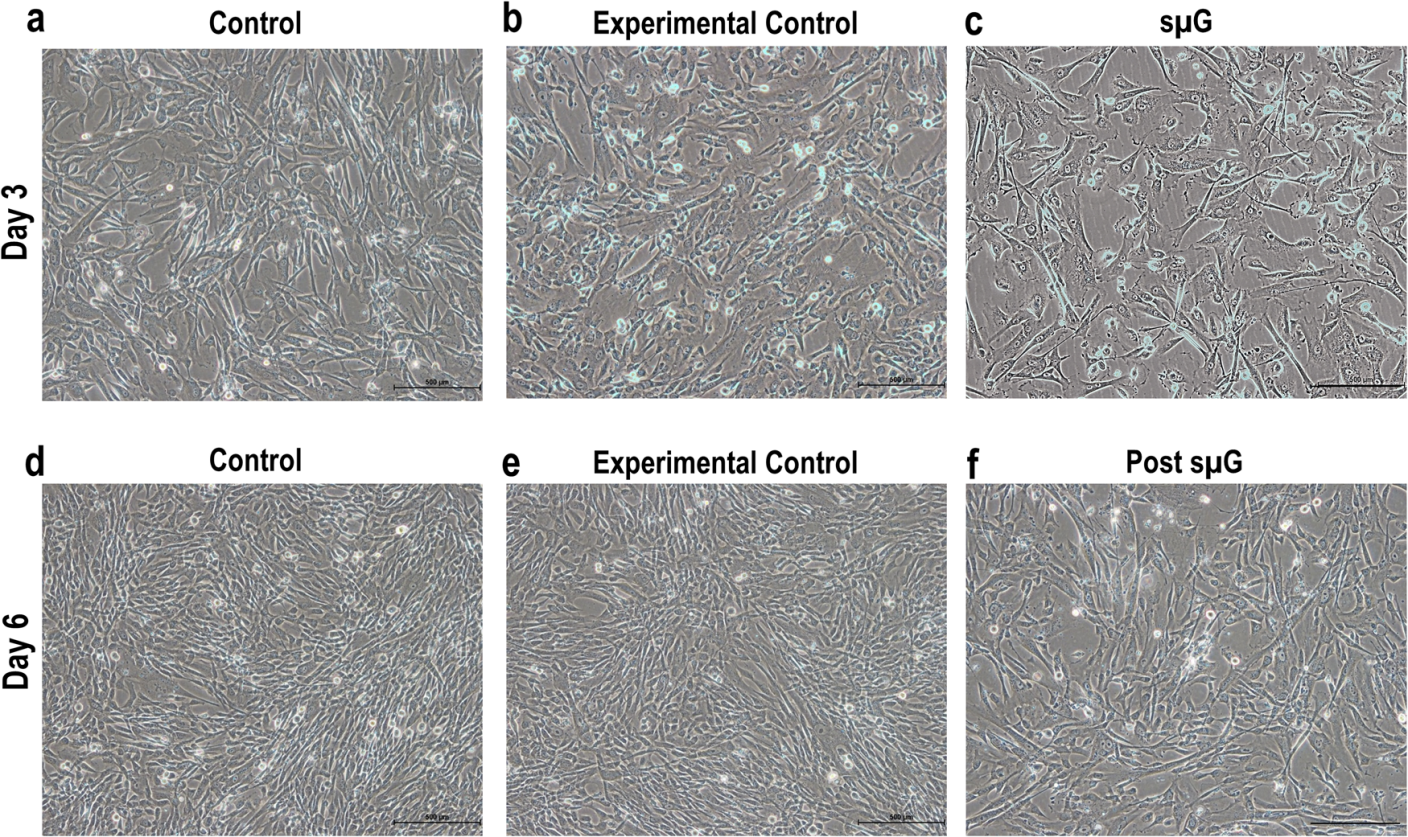
Phase contrast microscopic images of hWJSCs exposed to sµG and post-sµG conditions. hWJSCs cultured under sµG conditions were attached and growing on the tissue culture-treated flask with mitotic cells (**c**). hWJSCs cultured under sµG (**c**) and post-sμG (**f**) retain their short fibroblast-like phenotype like control (**a** and **d**) and experimental control (**b** and **e**) arms. Magnification ×200.

hWJSCs cultured under simulated lunar microgravity (sµG) were attached and grew normally on the tissue culture-treated flask with mitotic cells. The morphology of hWJSCs exposed to sµG and those observed after returning from microgravity conditions had no overt morphological differences, retaining their characteristic short fibroblast-like phenotype, similar to the control groups (Fig. 1a–f).

### Cell numbers

**Fig. 2:**
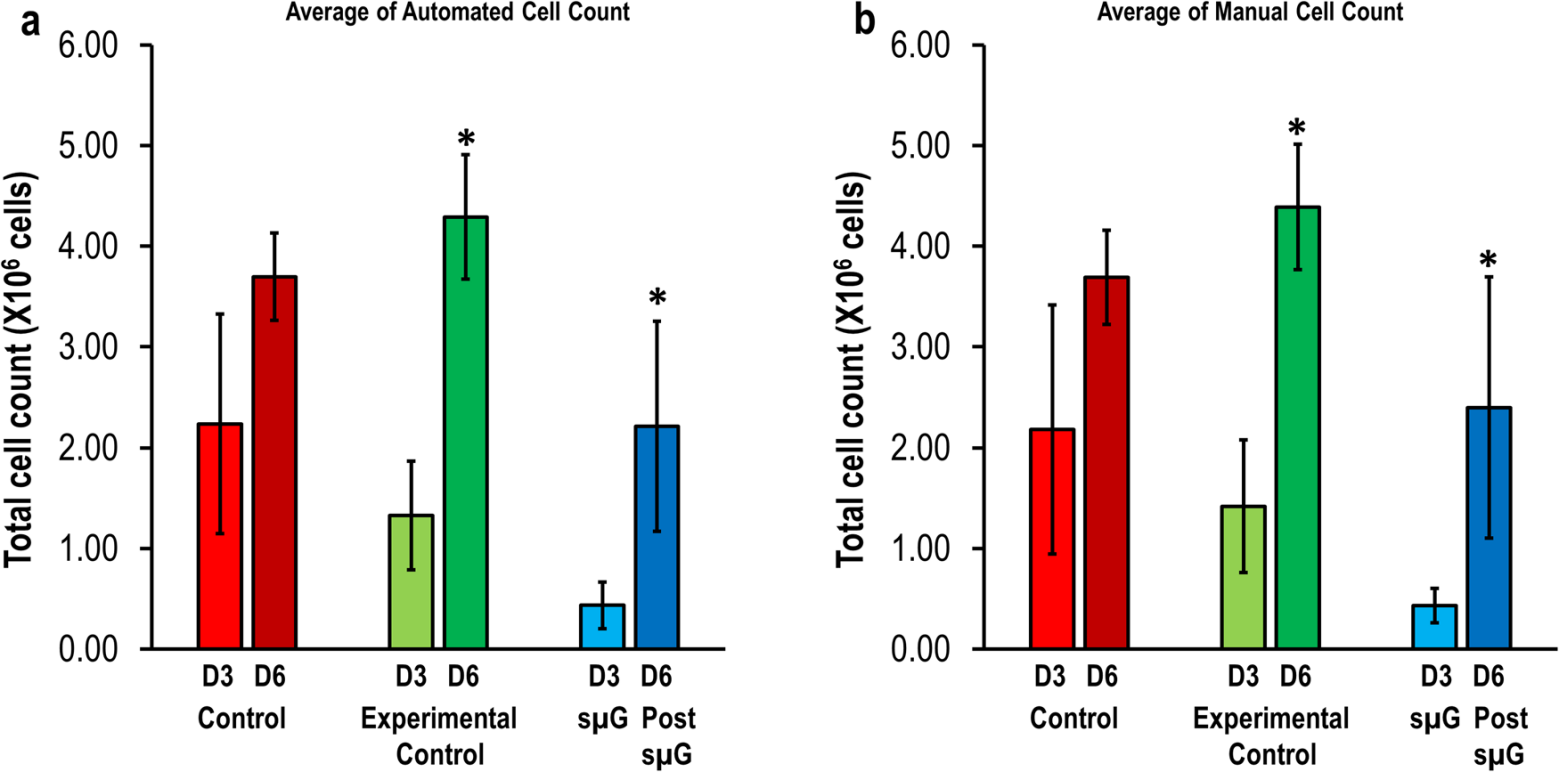
Trypan blue live cell counts for hWJSCs exposed to sµG and post-sµG conditions. Trypan blue live cell counts by automated (**a**) and manual (**b**) methods showed that the viable cell numbers were significantly decreased for hWJSCs exposed to sµG and post-sµG compared to control and experimental control arms. The cell numbers were increased for hWJSCs in the post-sµG compared to sµG (**a** and **b**). Statistical tests were performed using one-way ANOVA (*n* = 7). All values represent the mean ± SD of at least five independent experiments. *P* < 0.05 was statistically significant.

Trypan blue live cell counts by automated (Fig. 2a) and manual (Fig. 2b) methods showed that hWJSCs exposed to sµG for 3 days had statistically significant decreased cell numbers as compared to the control and experimental control arms. All the groups had increased cell numbers after returning to normal gravity (Fig. 2a, b).

### CD marker profile of hWJSCs

**Fig. 3:**
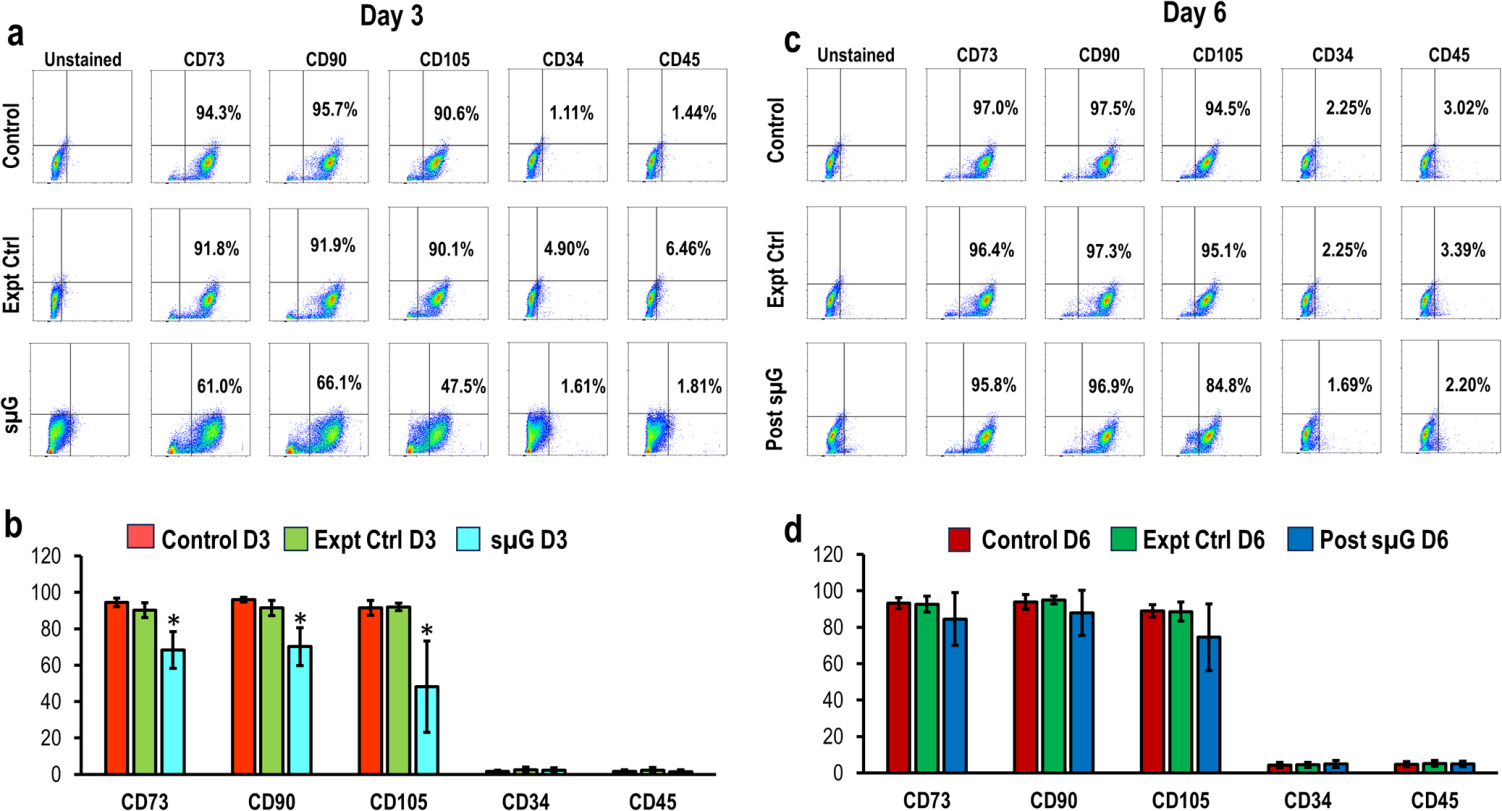
Flow cytometry analysis for CD marker profile of hWJSCs exposed to sµG and post-sµG conditions. CD marker percentages (**a** and **c**) for CD73, CD90, and CD105 decreased significantly in hWJSCs exposed to sµG compared to control and experimental control arms. **c** and **d** CD marker percentages for CD73, CD90, and CD105 were increased in hWJSCs exposed under post-sµG to levels similar to those recorded in the control and experimental control treatments. Statistical tests were performed by one-way ANOVA (*n* = 7). All values represent mean ± SD of at least five independent experiments. *P* < 0.05 was statistically significant.

FACS analysis of hWJSCs exposed to microgravity showed reduced CD marker profiles at day three. The percentage of positive CD markers experimental arm reduced to (mean ± SD) 68.38 ± 10.15% for CD 73; 70.25 ± 10.39% for CD90, and 48.21 ± 25.05% for CD105 compared to the control (94.46 ± 2.32% for CD 73; 96.02 ± 1.32% for CD90 and 91.46 ± 4.02% for CD105) and experimental control (90.24 ± 4.115% for CD 73; 91.42 ± 4.20% for CD90 and 92.00 ± 2.00% for CD105). There were no significant differences observed for CD34 and CD45 in any group (Fig. 3a, b). FACS analysis of post-sµG showed that the CD marker profiles were restored to normal in the sµG group (Fig. 3c, d), with no significant differences observed between groups.

### Apoptosis analysis

The hWJSCs from control, experimental control, and sµG exposures were analyzed for apoptosis using annexin V-FITC. Annexin V-FITC staining showed low percentages of positive cells for control (0.19%) and experimental control (0.19%). hWJSCs exposed to sµG had a higher percentage (2.0%) of positive cells compared to controls (Supplementary Fig. 1a–d).

### Bulk RNA sequencing analysis

#### Microgravity gene expression

**Fig. 4:**
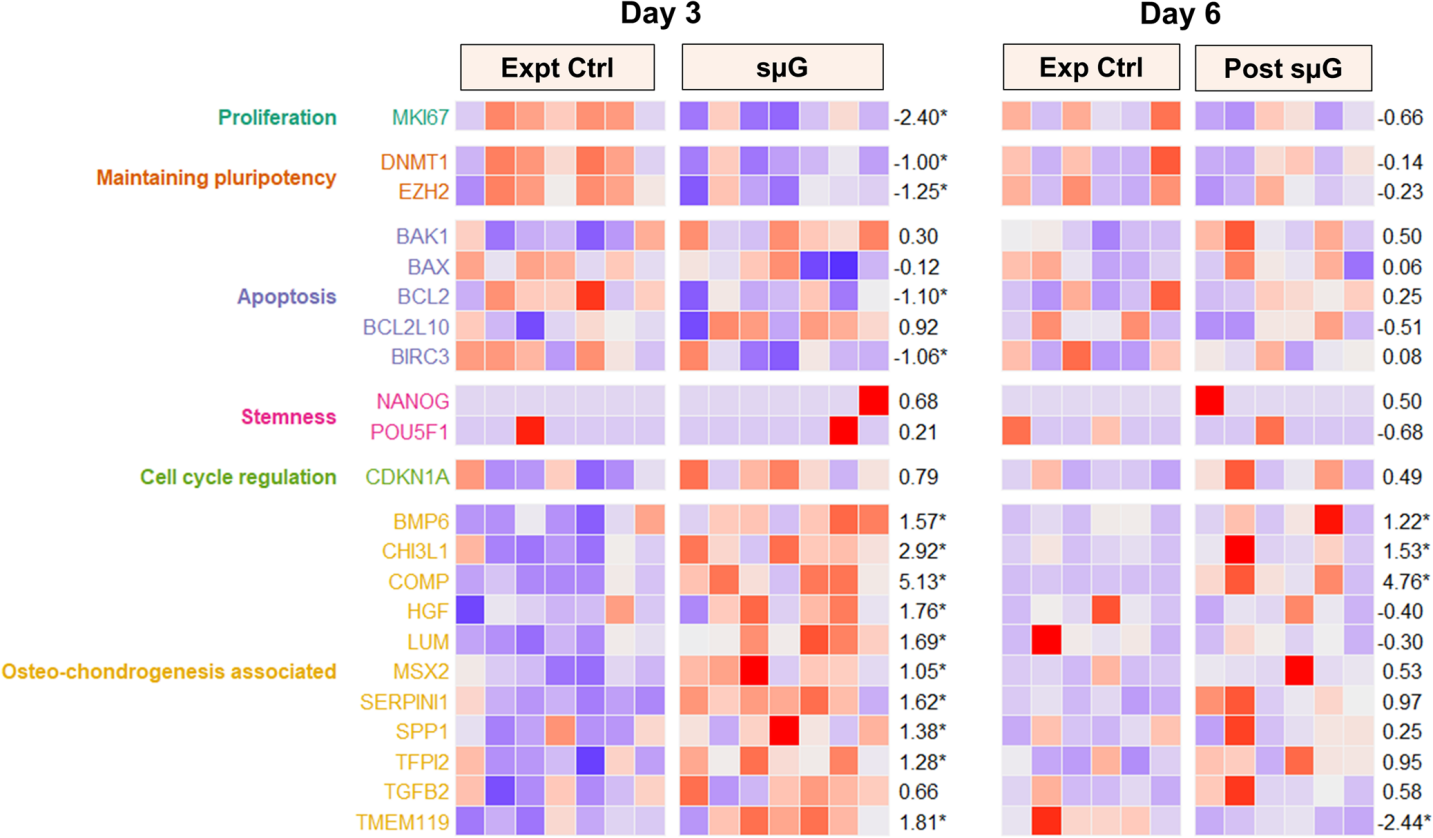
RNASeq analysis of hWJSCs exposed to sµG and post-sµG conditions. Heatmap of selected gene expression of Day 3 (experimental control, sµG) and Day 6 (experimental control, post sµG) hWJSCs. The LFC column represents the log2 fold change difference between sµG and experimental control (Day 3) or the post sµG and experimental control (Day 6). The asterisk symbol (*) after the LFC highlights genes that are statistically significant at transcriptome-wide analysis (at least 2-fold change and FDR < 5%).

A total of 636 differentially expressed genes (DEGs) with at least 2-fold change and FDR < 0.05 were identified (Supplementary Fig. 2a), with 308 significantly upregulated (Supplementary Table 1) and 328 significantly downregulated (Supplementary Table 2) in the sµG arm compared to experimental control. Cell proliferation gene *MKI67* was significantly downregulated (fold change = −2.40; FDR = 0.0000143). No significant differences were identified for the potent inhibitor of cell cycle progression *CDKN1A* in sμG, compared to experimental control. In terms of anti-apoptotic and pro-apoptotic-related genes, *BCL2* and *BIRC3* were significantly downregulated, whereas no significant difference observed for *BAX*, *BCL2L10,* and *BAK1* were detected in the sμG compared to experimental control. Genes essential for determining cell fate specification, including *DNMT1* and *EZH2,* were significantly downregulated in the sµG group, but there was no significant difference for embryonic stem cell pluripotent genes *POU5F1* and *NANOG* compared to experimental control. Interestingly, osteo-chondrogenesis-associated genes such as *SERPINI1, MSX2, TFPI2*, *BMP6, COMP, TMEM119, LUM, HGF, CHI3L1,* and *SPP1* were significantly upregulated in the sµG group compared to experimental control (Fig. 4). Changes in gene expression identified via RNA sequencing were confirmed with a panel of ten qPCR targets (*BAX*, *BCL2*, *CDKN1A*, *MKI67*, *NANOG*, *SOX2*, *BMP6*, *CHI3L1*, *COMP*, *MSX2*). qPCR data for five of ten selected targets (Supplementary Fig. 3) were in agreement with expression changes identified in RNA sequencing data.

#### Post microgravity gene expression

A total of 398 DEGs with at least 2-fold change and FDR < 0.05 were identified (Supplementary Fig. 2b), with 180 significantly upregulated (Supplementary Table 3) and 218 significantly downregulated (Supplementary Table 4) in the post-sµG arm compared to experimental control. Among these DEGs, there were no significant changes for proliferation gene *MKI67*, the cell cycle progression gene *CDKN1A*, and anti-apoptotic and pro-apoptotic related genes *BAX*, *BCL2*, *BIRC3*, *BCL2L10*, and *BAK1* in sμG after returning to normal gravity. There were no significant changes for pluripotent genes *(NANOG* and *POU5F1)* and cell fate- related genes (*DNMT1* and *EZH2)* in the post-sμG compared to the experimental control. Finally, osteo-chondrogenesis-associated genes such as *BMP6, COMP*, and *CHI3L1* remained significantly upregulated post-sμG, without changes when compared with their condition under sμG. However, *TMEM119* was significantly downregulated in the post-sμG, which was significantly upregulated in the sµG group (Fig. 4). Changes in gene expression identified via RNA sequencing were confirmed with a panel of ten qPCR targets (*BAX*, *BCL2*, *CDKN1A*, *MKI67*, *NANOG*, *SOX2*, *BMP6*, *CHI3L1*, *COMP*, *MSX2*). qPCR data for 6 of 10 selected targets (Supplementary Fig. 3) were in agreement with expression changes identified in RNA sequencing data.

### Pathway expression analysis

#### Microgravity gene expression

GSEA results are shown in Supplementary Tables 5 and 6. sµG-exposed cells had a total of 63 KEGG pathways with statistically significant positive normalized enrichment scores (NES), and these included oxidative phosphorylation, steroid hormone biosynthesis, sphingolipid metabolism, N-glycan biosynthesis, glycosaminoglycan biosynthesis (heparin sulfate and chondroitin sulfate), and glycerophospholipid metabolism. Likewise, 25 pathways had statistically significant NES and these included spliceosome, DNA replication, cell cycle, and nucleocytoplasmic transport. The top 20 pathways for sµG cells are shown in Supplementary Fig. 4.

#### Post-microgravity gene expression

After a further 72 h of 1.0 G reloading, sµG-exposed cells had a total of 98 pathways with statistically significant positive NES, and these included oxidative phosphorylation, sphingolipid metabolism, glycosaminoglycan biosynthesis (chondroitin sulfate) and the HIF-1 signaling pathway. Only 7 pathways had statistically significant negative NES. The GSEA plot and heatmap of the genes in the core enrichment in the oxidative phosphorylation for sµG-exposed cells and post-sµG-exposed cells are shown in Supplementary Fig. 5.

### Von Kossa staining

hWJSCs from control, experimental control and sµG exposures were cultured in the presence of osteogenic medium for 14 days. hWJSCs previously exposed to sµG for a period of 72 h had the highest number of von Kossa-stained cells compared to control and experimental control (Supplementary Fig. 6a–c). Manual counts (*n* = 6 wells/exposure) for positively-stained cells were (mean ± standard deviation) 17.8 ± 2.3, 7.7 ± 1.2, and 38.7 ± 3.3 for control, experimental control, and sµG treatments, respectively. The difference in positively stained cells between sµG and control and sµG and experimental control treatments was statistically significant (*p* < 0.001).

### Alcian blue stain

hWJSCs from control, experimental control, and sµG exposures were cultured in the chondrogenic medium. hWJSCs previously exposed to sµG had a higher number of cells that were positive for Alcian blue staining compared to compared to cells from control and experimental control exposures (Supplementary Fig. 6d–f). Manual counts (*n* = 6 wells/exposure) for positively stained cells were (mean ± standard deviation) 27.2 ± 5.9, 31.7 ± 3.3, and 54.3 ± 7.7 for control, experimental control, and sµG treatments, respectively. The difference in positively stained cells between sµG and control and sµG and experimental control treatments was statistically significant (*p* < 0.001).

### qRT-PCR analysis of osteocyte and chondrocyte-related genes

qRT-PCR analysis confirmed that hWJSCs from control, experimental control, and sµG groups readily differentiated into osteocyte and chondrocyte lineages when cultured using the respective differentiation media. The expression levels of the osteocyte-related genes, including bone sialoprotein (*BSP*), alkaline phosphatase (*ALP*), and osteocalcin (*OCN*), were significantly higher in sµG (3.12 and 6.63 fold) compared to control and experimental control arms, respectively (Supplementary Fig. 7a). Similarly, the expression levels of the chondrocyte-related genes such as cartilage oligomeric matrix protein (*COMP*), fibromodulin (*FMOD*), and collagen type II (*COL2A1*) were significantly higher in sµG (2.27 and 9.16 fold) compared to control and experimental control arms, respectively (Supplementary Fig. 7b). There were no significant differences in the expression levels for the osteogenic and chondrogenic-related genes between the control and experimental control (Supplementary Fig. 7a, b).

## Discussion

The primary findings of the study were that simulated lunar microgravity induced transient changes in the growth (only a modest increase in apoptosis marker Annexin V in sµG cells) and lineage differentiation of hWJSCs. Importantly, we show that with gravity re-loading, the population characteristics (both growth and stem cell markers) of sμG-exposed hWJSC rapidly return towards baseline, although several key lineage differentiation changes observed at transcriptional and protein levels were maintained.

Histological assessments of osteogenic (von Kossa) and chondrogenic (Alcian Blue) proteins in hWJSCs cultured in differentiation media showed statistically significant increased staining distribution in cells exposed to sµG, relative to 1.0 G experimental controls. These observations of accelerated differentiation in sµG-exposed cells are consistent with the pro-osteocyte–chondrocyte transcriptional changes observed in our DEG and GSEA analyses.

In this study, we examined the growth behaviors of hWJSCs, which encompassed observations of morphological changes, cell counts, the profile of MSC CD markers, and the identification of DEGs and enriched pathways through RNAseq analysis. Several previous reports have shown that simulated microgravity (sμG) had clear impacts on various stem cells^14^, including human embryonic stem cells (hESCs)^15,16^, human hematopoietic stem cells (hHSCs)^17,18^, and human mesenchymal stem cells (hMSCs)^7,19,20^. However, to the best of our knowledge, this is the first report to comprehensively describe the effects of simulated microgravity on hWJSCs by focusing on growth, stemness properties, and transcriptional profiling under sμG conditions and then after gravity re-loading.

Work by Quynh Chi and colleagues^12^ identified growth and cytoskeletal changes (filamentous actin organization) after a 72 h sμG treatment. Our data extends on this work, showing that alterations in growth return to baseline after 1.0 G reloading. Rather than focusing on the cytoskeletal arrangement and nuclear morphology, in the present study, we have taken an unbiased approach to identify changes at a transcriptional level in hWJSCs under and post- sμG. We have then correlated these findings with FACS and histological assessments of lineage markers and differentiation. A key point of interest is the maintenance of enhanced lineage differentiation in sμG exposed cells returned to 1.0 G. An additional interesting difference between our work and that of earlier studies is a lack of apparent difference in cell morphology. Quynh Chi et al. identified changes in cytoskeletal microtubule organization but not nuclear morphology. Additional studies have reported that human bone marrow MSCs (hBMMSCs) underwent a morphological transformation from spindle-shaped to rounded cells after exposure to sμG for 3 days^21^. As noted, our findings indicate that hWJSCs did not exhibit significant morphological changes when exposed to sμG and in the post-sμG environment. From our transcriptional data, key cytoskeletal components such as vimentin, actions, and tubulins were not differentially regulated. It is tempting to speculate that this observed phenomenon was due to differences in the type of hWJSCs used, although further work is required to substantiate this point.

It is well known that stem cells are very sensitive to their local environment^13^. In this study, trypan blue live cell counts showed that hWJSCs under sμG and post-sμG conditions had significantly decreased in cell numbers compared to their respective controls. Nonetheless, the cell numbers were significantly increased when the cells were returned to the normal culture condition (post-sμG) compared to sμG. Therefore, our data also suggest that the sμG environment could potentially stimulate rapid, reversible response to changes in cell cycle progression by inhibiting the proliferation rate of hWJSCs^13,22^. However, the mechanism underlying the reduction in cell growth is still poorly understood.

One possible explanation is that hWJSCs exposed to acute periods of microgravity experience a transient senescent state. Previous studies on hWJSCs have demonstrated that, following initial gene adaptations during the early hours of simulated microgravity, there is a decline in proliferative capability, likely associated with the onset of a molecular senescence program^7^. On the other hand, it has also been suggested that the impaired proliferation could be partly due to the regulation of the cytoskeleton in response to sμG^7^. Pala et al. observed that hWJSCs in μG overexpressed β-actin in the first hour, but then it was downregulated, suggesting that microgravity may reduce the cellular scaffold of the cytoskeleton, impairing the capacity of proliferation^7^. Our data suggest that, upon returning to normal conditions, these growth-slowing changes are resolved, leading to restored proliferative potential.

Our results also showed that the hWJSCs expressed MSC CD markers such as CD73, CD90, and CD105 for control, whereas their expression was significantly reduced in the hWJSCs after sμG environment exposure. These data suggest that hWJSCs were losing their stemness properties under sµG. A previous study that investigated bone marrow MSCs in a microgravity setting aboard the International Space Station did not report a difference in the triple-positive markers (CD73+, CD90+, CD105+) and had comparable proliferation rates. The differences with our results may be attributable to variations between the two cell types, as well as differences in the experimental design; this study involved a longer duration of exposure to different gravity exposures (~10^-6^ G at the International Space Station as opposed to simulated 0.16 G lunar conditions). Interestingly, in our model, when hWJSCs were returned to 1.0 G loading, the cells expressed all three MSC markers (CD73, CD90, and CD105) in a similar pattern as seen in their respective controls. The fact that both the CD markers and proliferation, which are affected in our model, returned to normal values after 72 h of exposure suggests that at least these capabilities are modifiable and that either (i) the cells have the ability to recover when the conditions to which they were subjected are normalized; or (ii) the undifferentiated component of the hWJSC population is able to continue a normal pattern of growth despite transient sμG exposure.

The reduced cell counts and decreased expression of MSC CD markers in our results align well with the observations derived from the RNA sequencing data. We have shown that there is a downregulation of the proliferation gene *MKI67* under sμG conditions. Furthermore, we reported a significant downregulation of the anti-apoptotic genes *BCL2* and *BIRC3*, while there were no changes observed for pro-apoptotic gene *BAK1*. These findings are broadly consistent with only a small change in apoptotic cells between control and sμG-exposed hWJSC as determined by our Annexin V analysis. Interestingly, others have also reported downregulation of *BCL2* after extended exposure to microgravity^7^, providing further insight into the characteristics of these cells under microgravity conditions. These findings are consistent with previous reports indicating that microgravity may induce alterations in cell cycle progression, leading to inhibited cell proliferation^12,22^.

We showed that genes essential for determining stem cell fate (*DNMT1 and EZH2*) were significantly downregulated under sμG, whereas there were no changes for post-sμG exposures. These results are consistent with other studies that have also demonstrated *OCT4* and *NANOG* expression is downregulated under microgravity^7^. These findings suggest that hWJSCs under an sμG environment may differentiate into specific lineage states due to the reduction/loss of cell fate mediators and that these changes are permanent and do not revert to normality after gravity is restored. Additional studies employing single-cell sequencing approaches will be required to confirm the stable presence of divergent cell populations following transient sμG exposures.

The loss of pluripotency and the shift towards a more definitive differentiation state associated with microgravity exposure was observed at the level of gene expression and immunohistochemical staining. Our RNAseq DEG analysis suggests that sµG can induce prechondro-osteoblast differentiation of hWJSCs. GSEA analyses showed significant enrichment of upregulated transcripts in pathways related to osteocyte–chondrocyte lineages. For example, Shum and colleagues reported that bone marrow mesenchymal stem cells activated oxidative phosphorylation during osteogenic differentiation^23^. Increases in chondroitin sulfate (a key cartilage glycosaminoglycan) were reported in the bone marrow and synovial mesenchymal stem cells undergoing chondrogenic differentiation^24^. Similarly, heparin sulfate is reported as being an important regulator of signaling pathways during chondrogenesis^25^. Sphingolipids (including Sphingosine-1-phosphate) are implicated in a range of osteogenesis-related processes, including cell differentiation^26^. The enrichment of HIF-1 signaling in post-sμG hWJSc may be consistent with the alleviation of sμG-induced osteogenic differentiation (Palomaki and colleagues report that osteogenic differentiation of mesenchymal stem cells from bone marrow was accompanied by a reduction in HIF-1α mRNA)^27^. Interestingly, we have observed that the expression of osteo-chondrogenesis-associated genes, including osteogenic differentiation predictive marker genes (*HGF, TFPI2* and *SERPINI*)^28^, osteogenesis promoting genes (*BMP6* and *MSX2*)^29,30^, extracellular matrix remodeling genes (*SPP1*, *CHI3L1*, *LUM*)^31–33^ and cartilage-specific gene (COMP)^34^ were upregulated under sμG conditions and that a smaller number of these transcripts remained differentially expressed (notably *BMP6, CHI3L1, COMP*) once 1.0 G loading was restored.

Oxidative phosphorylation plays an important role in both stemness maintenance and differentiation for stem cells. It has been shown that sμG inhibits oxidative phosphorylation and suppresses osteogenic differentiation in rat MSCs^19^. However, our current study showed that sμG activated oxidative phosphorylation under both sμG and post-sμG conditions. Therefore, although speculative, our data suggest that sμG may induce osteo-chondrogenesis of hWJSCs through activation of oxidative phosphorylation. Alternatively (and in the absence of mechanistic data), it is also possible that observed changes in oxidative phosphorylation are associative rather than causal in terms of changes in cell fate^19^.

Of additional interest is the observation that our findings in hWJSCs seem to be distinctive to this specific type of MSC. Using hBMSCs, two groups have studied the RNAseq expression of this cell type under 1.0 G and simulated microgravity conditions, reporting that osteogenic differentiation was severely hindered after 7 and 12 days^35,36^, respectively. Although the disparity in results could be attributed to the duration of exposure to microgravity, wherein longer durations may lead to greater stress stimuli that result in decreased expression of these differentiation genes, it is also important to consider that MSCs from different sources have distinct characteristics and properties. Future studies will need to clarify whether longer exposures to hWJSCs exert changes different from those observed in our study.

In summary, our results suggest that simulated lunar microgravity significantly reduces cell proliferation, modifies MSC CD markers, and affects the stemness features of hWJSCs. These changes in cell growth appear to be reversible; however, the differentiation due to the loss of stemness could be more permanent—as shown by the maintenance of some osteocyte–chondrocyte markers and increased von Kossa and Alcian Blue staining in sμG hWJSCs cultured at 1.0 G in differentiation media. Therefore, our findings support the conclusion that a simulated lunar microgravity environment could play a role in enhancing the osteo-chondrocyte lineage differentiation of hWJSCs, with potential clinical and biotechnique implications for the use of these cells.

## Methods

### Isolation and propagation of human Wharton’s jelly stem cells (hWJSCs)

Human umbilical cords (UC) were obtained with written informed patient consent and approval from the Singapore Ministry of Health, Domain Specific Review Board (DSRB). The hWJSCs were derived from human umbilical cords according to our previously published protocol^37,38^. Briefly, the umbilical cord was cut into 1–2 cm sections and then dissected lengthwise. Sections were placed with their inner surface faced down into DMEM medium (Invitrogen) containing an enzymatic solution comprised of 2 mg/ml collagenase type I (Invitrogen Life Technologies, Carlsbad, CA), 2 mg/ml collagenase type IV (Invitrogen) and 100 IU of hyaluronidase (Invitrogen) in 100 mm sterile plastic dishes (Becton Dickinson, BD, NJ, USA) and incubated at 37 °C in a 5% CO_2_-in-air atmosphere for 45 min. Subsequently, the enzymatic solution containing the Wharton’s jelly was transferred to sterile 15 ml tubes (BD), syringed through an 18-gauge needle to release the cells from the jelly before being centrifuged at 300×*g* for 10 min. The supernatant was then decanted, and the cell pellets were resuspended in an hWJSC culture medium comprised of 80% DMEM high glucose supplemented with 20% fetal bovine serum (FBS), (Biochrom, Berlin, Germany), 1% non-essential amino acids, 2 mM l-glutamine, 0.1 mM β-mercaptoethanol, 1% insulin–transferrin–selenium (ITS), antibiotic–antimycotic mixture (Invitrogen) and 16 ng/ml basic fibroblast growth factor (bFGF) (Millipore Bioscience Research Agents, Temecula, CA).

### Experimental setup for simulated lunar microgravity (sµG)

#### Simulated lunar microgravity

hWJSCs (*n* = 7) were seeded at a density of 5.0 × 10^5^ cells in T25-tissue culture-treated flasks with vented-caps for the control arm, occluded caps for experimental control and sµG arms. For the control arm, cells were seeded with 5 ml of hWJSC medium and incubated at 37 °C in a 5% CO_2_-in-air atmosphere for three days (with CO_2_ gas exchange). For both experimental control and sµG arms, the flasks were completely filled with hWJSC culture medium to avoid air bubbles and shear stress during rotation, which is necessary for sµG. The flasks were tightly sealed with parafilm to prevent any media from leaking and incubated in the same 37 °C incubator as the control arm cells for three days. The sµG flasks were taped onto the rotating platform of a Random Positioning Machine (RPM; Yuri Gravity, Meckenbeuren, Germany), which was placed in the CO_2_ incubator and run on a pre-programed protocol to stimulate lunar gravity (0.16 G) for 3 days. Each biological replicate (*n* = 7) was cultured in triplicates that were pooled for analyses.

#### Post microgravity

After 3 days, hWJSCs (*n* = 7) were disassociated and seeded at a density of 5 × 10^5^ cells in T25-tissue culture flasks with vented caps for all arms; control, post-experimental control and post-experimental (post- sμG). Across the board, cells were cultured with 5 ml of hWJSC medium and incubated at 37 °C in a 5% CO_2_-in-air atmosphere for another 3 days (with CO_2_ gas exchange). Each biological replicate (*n* = 7) was cultured in triplicates that were pooled for analyses.

### Cell morphology

Cell attachment, morphological changes, and growth of hWJSCs exposed to microgravity and post-microgravity were monitored and photographed using inverted phase contrast optics (Nikon Instruments, Tokyo, Japan).

### Trypan blue vital counts

The viability of hWJSCs cultured under sµG and post microgravity were quantified using trypan blue vital counts. An aliquot of each cultured hWJSCs from control, experimental control, and sµG arms at day 3 (sμG) and day 6 (post-sμG) was taken and stained with 0.4% Trypan Blue (vital dye) (Sigma) for 1 min at room temperature. The numbers of live cells (unstained) were either counted manually using a hemocytometer (Hausser Scientific, Horsham, PA, USA) or automatedly using the Luna Automated Cell Counter (Bio Cat, Heidelberg, Germany).

### Fluorescence-activated cell sorting (FACS) analysis

hWJSCs were disassociated using TrypLE^TM^ Express (Invitrogen) for 3–5 min prior to PBS wash to obtain single-cell suspensions, which were then blocked with 10% normal goat serum (NGS) (Invitrogen Life Technologies, Carlsbad, CA) for 30 min to prevent non-specific binding. The cells were incubated with primary antibodies: CD34, CD45, CD73, CD90, and CD105 (1:100, Biolegend, San Diego, CA) for 1 h followed by incubation with Alexa Fluor^®^488 (1:500) secondary antibody (Invitrogen Life Technologies, Carlsbad, CA) for 30 min. The cells were washed with PBS and re-suspended in 10% NGS. Finally, the cells were filtered using a 70 µm nylon strainer (BD Bioscience) to remove any cell clumps and then analyzed using a CytoFLEX LX Analyzer (Beckman Coulter, Fullerton, CA).

### Annexin V-FITC assay

The annexin V-FITC assay was carried out on hWJSCs from control, experimental control, and sµG exposures to evaluate rates of apoptosis. Briefly, the cells were dissociated with TrypLE^TM^ Express (Invitrogen), washed once with phosphate-buffered saline (PBS), and then with Annexin V binding buffer (1×). The cells were stained with 5 μl Annexin V-FITC (Promega) and counterstained with propidium iodide (1 μg/ml) (Promega) for 15 min at room temperature, then analyzed using a CytoFLEX LX Analyzer (Beckman Coulter, Fullerton, CA).

### RNA extraction

Total RNA was extracted from hWJSCs cultured under sµG and post-sµG conditions (including control, experimental control, and sµG arms) using the RNeasy Mini kit (Qiagen, Venlo, Netherlands). RNA quality and quantity were measured using a Nanodrop™ Spectrophotometer (Nanodrop Technologies, Wilmington, DW) and an Agilent 2100 Bioanalyzer using an Agilent 6000 Nano RNA kit (both Agilent, Waldbronn, Germany). 1000 ng of total RNA (minimum RIN value 9.0) in a 25 μl volume was submitted for sequencing.

### RNA sequencing

RNA-seq library was prepared (3’ directional, polyA enrichment) from total RNA with 150-bp paired-end sequencing on the NovaSeq 6000 system by NovogeneAIT Genomics Singapore. Samples were sequenced to a target depth of 30 million reads.

### RNA-seq data processing and quality control

The 150-bp paired-end sequenced reads were processed using the nf-core/rnaseq v3.10.1 pipeline^39^ with nextflow v22.10.4^40^. Briefly, raw reads were trimmed using Trim Galore! v0.6.7^41^ to remove low-quality bases and adapters. Trimmed reads were aligned to the human reference genome, hg38, with STAR v2.6.1d aligner^42^. The gene counts were generated using Salmon v1.9.0^43^ quantification of the aligned reads. Principle component analysis was conducted for quality control assessment.

### Bioinformatic analysis

All downstream analyses were performed using R statistical software version 4.2.1^44^. Differential gene expression analysis was performed using the DESeq2 package v1.36.0^45^, adjusting for cell line differences. Genes with at least 2-fold change in expression and FDR < 0.05 were considered significant. Results were visualized using the EnhancedVolcano v1.14.0^46^ and ComplexHeatmap v2.12.1^47^. Pathway enrichment analysis (GSEA) was run on all genes ranked by their signed log *p*-value using clusterProfiler v4.9.0^48^ and KEGG database^49^. Pathways with FDR < 0.10 were considered significant.

### Confirmatory studies

To confirm that the transcriptional changes identified in the initial sµG and post-sμG studies resulted in lineage shifts, we performed an additional set of experiments to induce osteogenic and chondrogenic differentiation in sµG hWJSCs and matched controls. As below, these cells were then assessed for lineage differentiation using quantitative PCR and immunohistochemistry. All osteogenic and chondrogenic differentiation studies were performed in triplicate.

#### Osteogenic differentiation

For the induction of osteogenic differentiation, hWJSCs from control, experimental control, and sµG were seeded (5.0 × 10^5^ cells/dish) in T25-tissue culture treated flasks and incubated at 37 °C in a 5% CO_2_-in-air atmosphere for 24 h to allow attachment. The medium was then switched to osteogenic medium containing DMEM supplemented 5% FBS, 0.17 mM l-ascorbic-acid (Sigma, MO), 100 nM dexamethasone (Sigma, MO), antibiotic-antimycotic mixture (Invitrogen) and 10 mM β-glycerophosphate (Sigma, MO) and the cells were cultured for 14 days with fresh changes of medium every 48 h.

#### Chondrogenic differentiation

For the induction of chondrogenic differentiation, hWJSCs from control, experimental control, and sµG were seeded (5.0 × 10^5^ cells/dish) in T25-tissue culture treated flasks and incubated at 37 °C in a 5% CO_2_-in-air atmosphere for 24 h to allow attachment. The medium was then changed to chondrogenic medium containing DMEM supplemented with 1% ITS, 0.17 mM l-ascorbic-acid, 100 nM dexamethasone, 1 mM sodium pyruvate, 0.35 mM proline (Sigma, MO), antibiotic–antimycotic mixture (Invitrogen) and 10 ng/ml TGFβ-3 (Sigma, MO) and the cells were cultured for 14 days with fresh changes of medium every 48 h.

#### Von Kossa staining

To assess hWJSCs' osteogenic potential, mineralization was evaluated by Von Kossa staining for hWJSCs cultured in an osteogenic medium for 14 days. Briefly, cells were washed with PBS and fixed in a 4% formaldehyde solution for 10 min at room temperature. The cells were then washed with distilled water and stained in 1% silver nitrate solution under UV light for 60 min. The cells were then washed again with distilled water, treated with 3% sodium thiosulfate for 5 min, and counterstained with 1% nuclear fast red for 5 min. The stained cells were washed with distilled water and photographed using inverted phase contrast optics (Nikon Instruments, Tokyo, Japan).

#### Alcian Blue staining

To evaluate hWJSCs chondrogenic potential, hWJSCs were stained with alcian blue staining for the cells cultured in a chondrogenic medium for 14 days. Briefly, the cells were washed and stained with 0.5% Alcian Blue (Sigma, St. Louis, MO) for 30 min at room temperature and then rinsed with tap water. The slides were counterstained with 0.1% Nuclear Fast Red (Sigma, St. Louis, MO) for 5 min and then photographed using inverted phase contrast optics (Nikon Instruments, Tokyo, Japan).

#### Quantitative real-time polymerase chain reaction (qRT-PCR)

RNA samples were transcribed to cDNA using Tetro cDNA Synthesis kit (Bioline, Eveleigh NSW, Australia). qRT-PCR analysis was performed using ABI PRISM 7500 Fast Real-Time PCR System (Applied Biosystems, Waltham, MA) using SYBR green master mix (Applied Biosystems). The final primer concentration in each reaction per well was 1 μM, and 20 ng of cDNA was used for each reaction well of 96-well plate. The cycling conditions were as follows: initial denaturation at 95 °C for 10 min, followed by 40 cycles of denaturation at 95 °C for 15 s, annealing at 60 °C for 30 s, and extension at 72 °C for 30 s. Glyceraldehyde-3-phosphate dehydrogenase (GAPDH) was used as the normalization control, and untreated respective samples were used as the calibrator. The relative quantification was performed using the comparative CT (2^−ΔΔCT^) method. The results were expressed as mean ± SD from three replicates for individual experiments.

### Supplementary information

Supplementary Information

## Data Availability

Source data is available in the supplementary section of the paper. Sequencing data deposited in GEO (GSE248366).
