# Supplementary material for: Simulated lunar microgravity transiently arrests growth and induces osteocyte-chondrocyte lineage differentiation in human Wharton’s jelly stem cells
Source: NPJ Microgravity. 2024 May 4;10:51. doi: 10.1038/s41526-024-00397-1 (PMC11069510; doi:10.1038/s41526-024-00397-1)
Supplement: Supplementary file 1 — Supplementary Information [file 41526_2024_397_MOESM1_ESM.pdf]

Simulated Lunar Microgravity Transiently Arrests Growth and Induces Osteocyte-Chondrocyte Lineage Differentiation in Human Wharton's Jelly Stem Cells.

Supplementary Figures

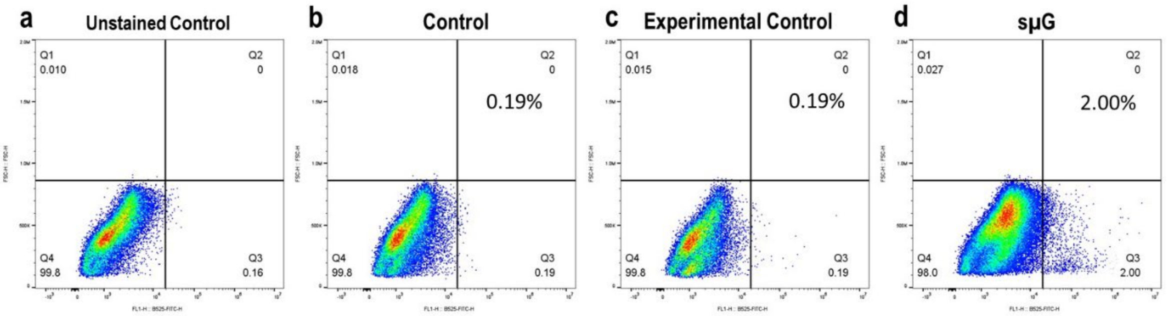

Supplementary Figure 1: Annexin V FITC analysis of hWJSCs exposed to sμG conditions. Annexin V FITC analysis. hWJSCs from control (**b**), experimental control (**c**) and sμG (**d**) showed low percentages of positive cells for Annexin V FITC stain.

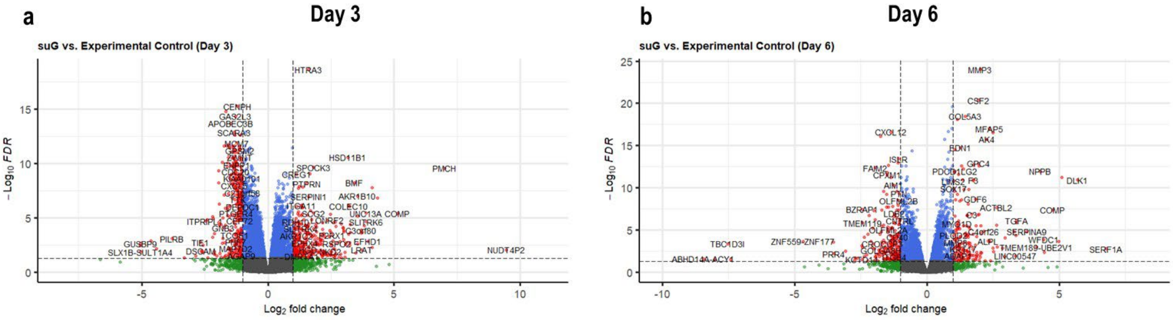

Supplementary Figure 2: Volcano plots representing differential gene expression for hWJSCs exposed to sμG and post-sμG conditions. The x-axis represents the biological significance ($\log_2$ fold change) and y-axis represents the statistical significance ($\log_{10}$ FDR). Dotted vertical lines represent the biological significance threshold (at least 2-fold change) and dotted horizontal lines represent FDR < 5% threshold.

NUS Confidential

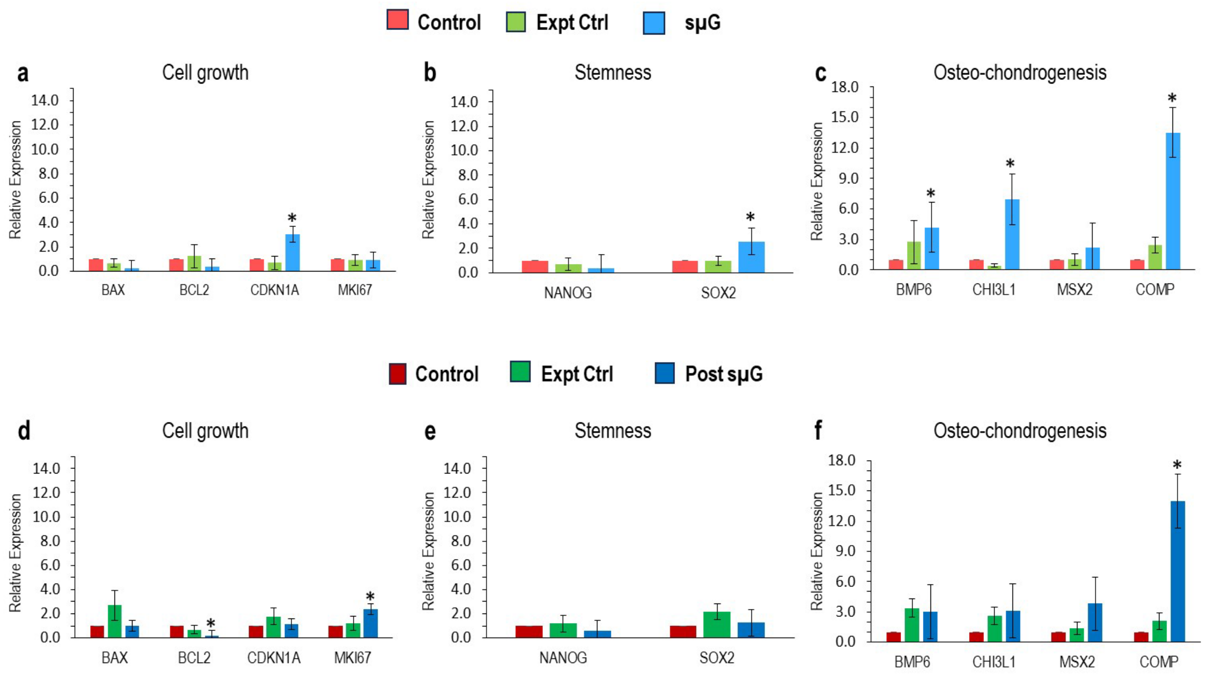

Supplementary Figure 3: qRT-PCR analysis of hWJSCs exposed to sμG and post-sμG conditions. Analysis of RNA transcript expression of apoptosis and cell cycle regulatory genes (**a&d**), cell proliferation genes (**a&d**), pluripotent geness (**b&e**) and Osteo-chondrogenesis associated geness (**c&f**) in hWJSCs exposed to sμG and post-sμG.. All values represent as mean ± SD of at least three independent experiments. $P < 0.05$ was statistically significant.

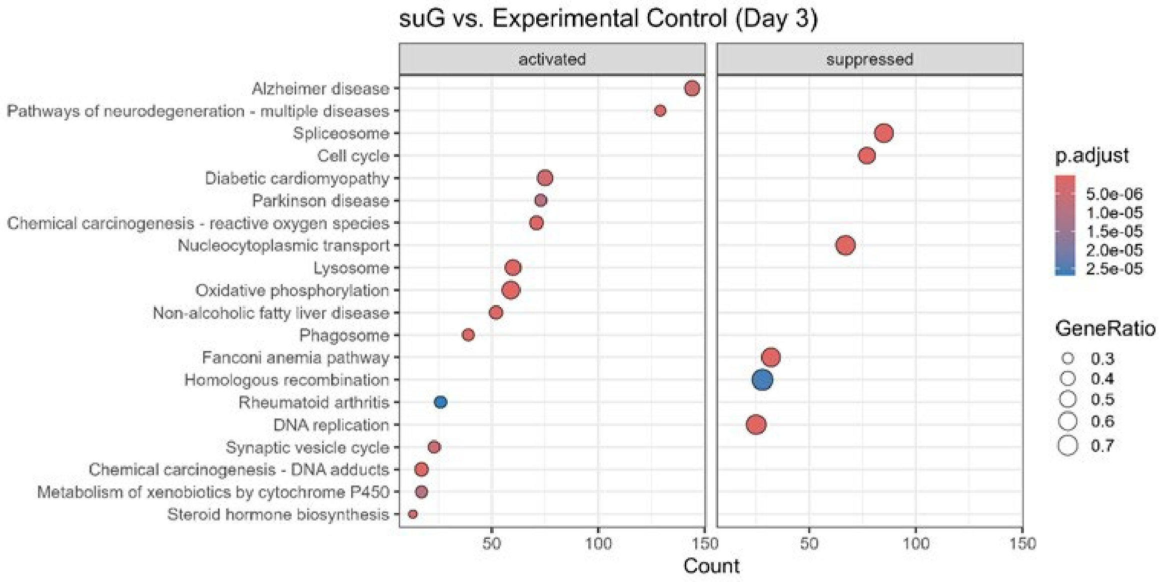

NUS Confidential

Supplementary Figure 4: Pathway enrichment analysis for hWJSCs exposed to sµG and post-sµG conditions. KEGG pathway analysis of the top 20 KEGG enriched gene pathways for sµG cells (experimental control, sµG).

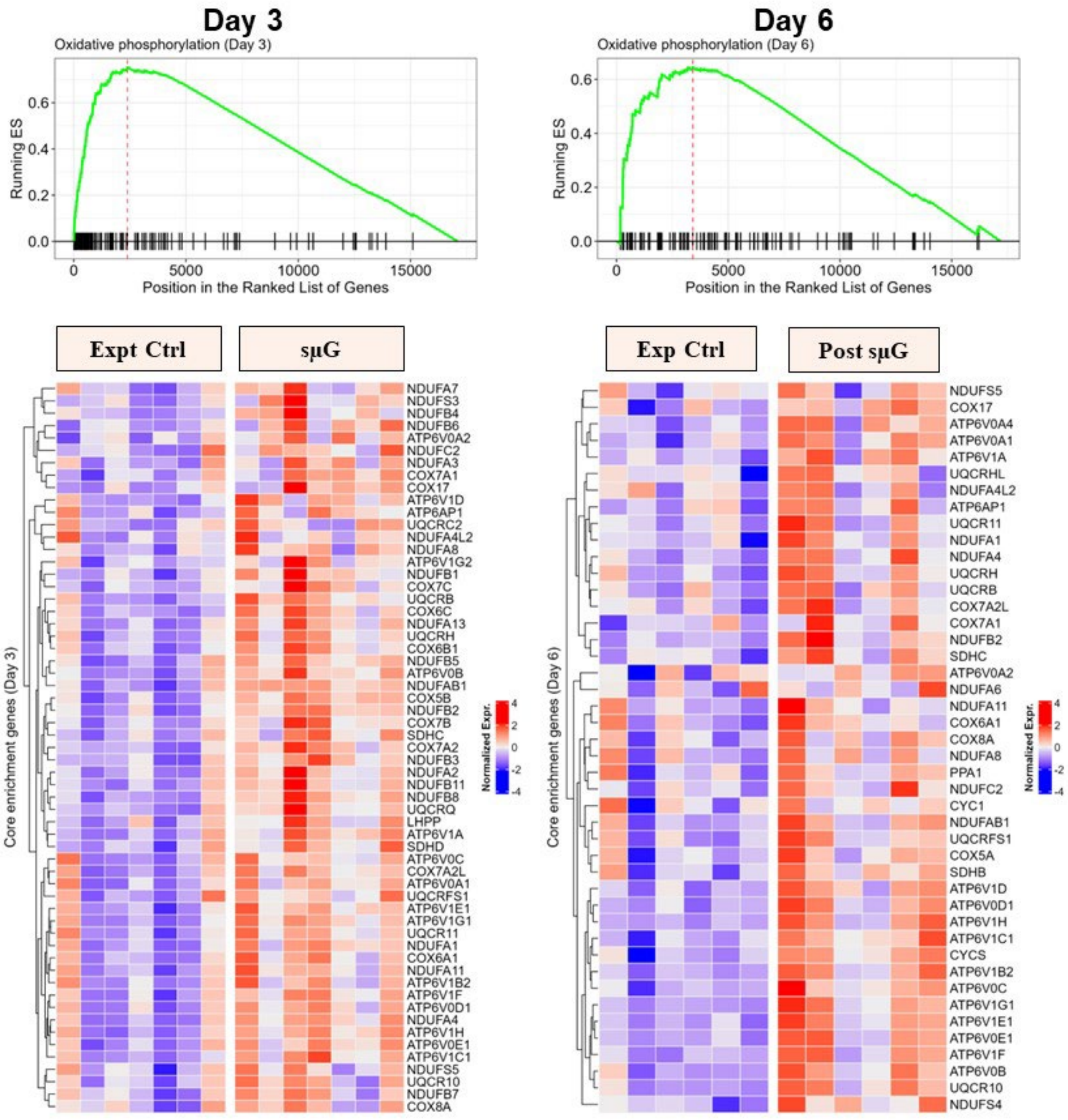

Supplementary Figure 5: Pathway Expression Analysis of hWJSCs exposed to sµG and post-sµG conditions. GSEA plots and heatmaps of the genes in the core enrichment for Day 3 (experimental control, sµG) and Day 6 (experimental control, post sµG) for the Oxidative Phosphorylation pathway.

NUS Confidential

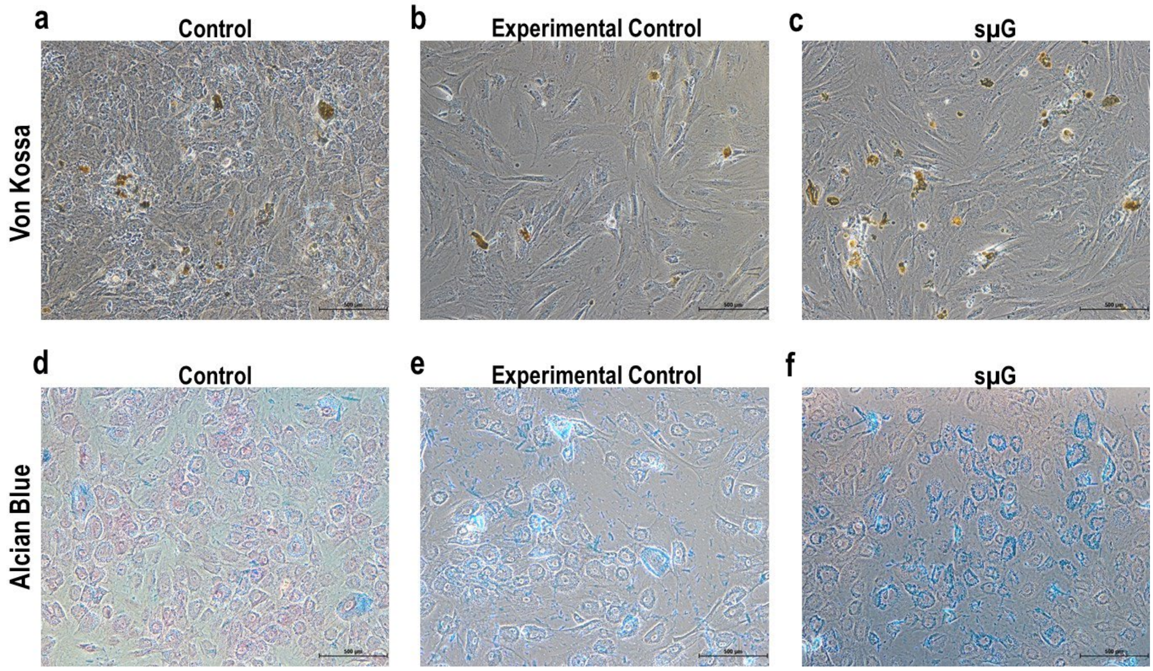

Supplementary Figure 6: Von Kossa (Osteogenic) and Alcian blue (Chondrogenic) staining of hWJSCs exposed to sμG conditions. The hWJSCs from each treatment arm were cultured in osteogenic and chondrogenic differentiation media, staining positive for Von Kossa (**a-c**) and Alcian blue stains (**d-f**). hWJSCs previously exposed to sμG had the greatest staining intensity for Von Kossa (**c**) and Alcian blue (**f**) compared to control **(a&d)** and experimental control (**b&e**). Magnification 100x.

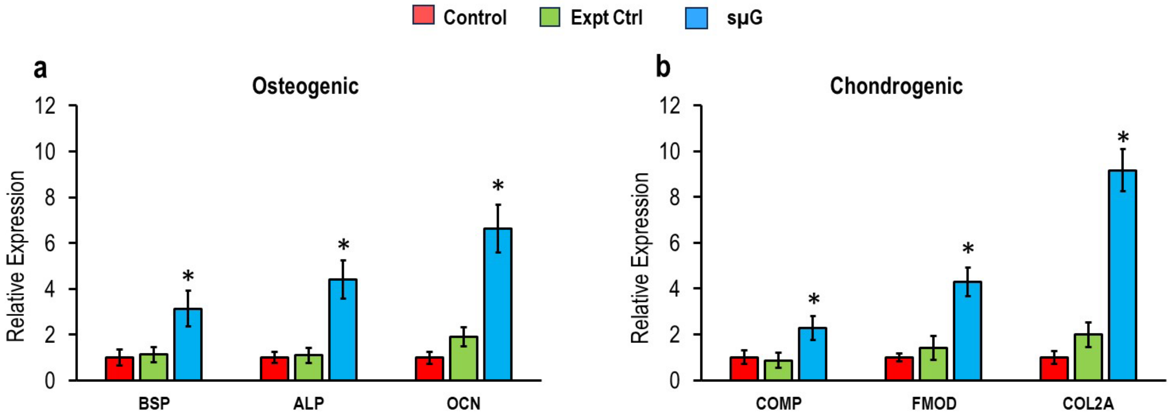

Supplementary Figure 7: qRT-PCR analysis for osteogenic and chondrogenic-related genes. The hWJSCs previously exposed to sμG had significantly upregulated expression of

NUS Confidential

osteocyte-related gene transcripts (BSP, ALP and OCN) and chondrocyte-related gene transcripts (COMP, FMOD and COL2A1) compared to cells from control and experimental control arms (**a-b**). There were no significant differences in the expression levels for the osteogenic and chondrogenic -related genes between control and experimental control (**a-b**). All values represent as mean ± SD of at least three independent experiments. $P < 0.05$ was considered statistically significant.

Supplementary Tables

Supplementary Table 1: sμG upregulated.

https://mynbox.nus.edu.sg/u/1rP0d5Gz9HrEs_R_/01f598de-1258-40ff-ac87-cdf046c578b8?l

Supplementary Table 2: sμG downregulated.

https://mynbox.nus.edu.sg/u/1rP0d5Gz9HrEs_R_/01f598de-1258-40ff-ac87-cdf046c578b8?l

Supplementary Table 3: Post sμG upregulated.

https://mynbox.nus.edu.sg/u/1rP0d5Gz9HrEs_R_/01f598de-1258-40ff-ac87-cdf046c578b8?l

Supplementary Table 4: Post sμG downregulated.

https://mynbox.nus.edu.sg/u/1rP0d5Gz9HrEs_R_/01f598de-1258-40ff-ac87-cdf046c578b8?l

Supplementary Table 5: sμG (Day 3)

https://mynbox.nus.edu.sg/u/1rP0d5Gz9HrEs_R_/01f598de-1258-40ff-ac87-cdf046c578b8?l

Supplementary Table 6: Post sμG (Day 6)

https://mynbox.nus.edu.sg/u/1rP0d5Gz9HrEs_R_/01f598de-1258-40ff-ac87-cdf046c578b8?l

NUS Confidential
